# ID 313 - Osimertinibe no Tratamento Oral de Primeira Linha do Câncer de Pulmão de Células Não Pequenas (CPCNP) Localmente Avançado na Perspectiva do Sistema Único de Saúde: análise de impacto orçamentário

**DOI:** 10.5327/2237-9622.2025.v34s1.313

**Published:** 2025-11-25

**Authors:** Suelem Tavares da Silva Penteado, Ana Isabella Arruda Meira Ribeira, Bruna Monteiro Rodrigues, Lauriston Medeiros Paixão, Virginia Kagure Wachira, Alexander Itria

**Keywords:** análise econômica, carcinoma pulmonar de células não pequenas, inibidores de tirosina quinase, *market share*

## Abstract

**Introdução:**

O câncer de pulmão é um dos mais agressivos, 70% dos casos são diagnosticados já na forma avançada ou metastática, sem opções de tratamento definitivo, o que contribui para o aumento de 90% da taxa de letalidade. Embora o Sistema Único de Saúde (SUS) tenha incorporado erlotinibe e gefitinibe para o tratamento do câncer de pulmão de células não pequenas (CPCNP) com mutação no receptor do fator de crescimento epidérmico (EGFR), são necessárias intervenções mais eficazes e seguras. Sendo assim, o objetivo deste estudo foi avaliar o impacto financeiro a ser onerado pelo Sistema Único de Saúde (SUS) caso o osimertinibe em monoterapia fosse incorporado para indicação terapêutica de primeira linha no tratamento do CPCNP em relação a outros representantes da classe de inibidores de tirosinoquinase já incorporados ao SUS (erlotinibe e gefitinibe) para tratamento dessa condição clínica.

**Método:**

Este estudo foi desenvolvido conforme as premissas das do Ministério da Saúde (Brasil, 2012). A análise foi realizada por demanda aferida associada ao método epidemiológico para estimar a população atendida com CPCNP localmente avançado ou metastático, sob a perspectiva do SUS, sendo considerado um horizonte temporal de cinco anos e cenários atual e alternativos (conservador e agressivo). Para o, foram considerados dois cenários alternativos: cenário alternativo 1 com taxa de difusão conservadora (aumento de 10% ao ano, chegando em cinco anos a atingir 50% dos pacientes). Além disso, foi estimado o cenário alternativo 2, prevendo uma taxa de difusão agressiva (aumento do uso de 20% ao ano, atingindo em cinco anos 90% dos pacientes). Com relação aos custos, a análise considerou os custos de aquisição dos medicamentos e de monitoramento (exames laboratoriais e de imagem).

**Resultados:**

A projeção do impacto orçamentário, ao longo de cinco anos com taxa de difusão conservadora, para pacientes sem tratamento prévio resultou em um incremento acumulado de R$ 786.946.282,73. Ao considerar uma difusão mais agressiva do osimertinibe para 90% dos pacientes elegíveis ao final de cinco anos, um total acumulado de R$ 1.557.945.251,69 foi observado. Portanto, a avaliação do impacto orçamentário de osimertinibe, nos cenários avaliados, mostrou-se oneroso para o sistema público de saúde. Apesar de bons resultados no quesito de eficácia e segurança em relação aos comparadores gefitinibe e erlotinibe, o custo de incorporação ainda é alto.

**Conclusão:**

Quando se compara a análise de impacto orçamentário do gefitinibe, que foi incorporado no SUS no ano de 2013, o custo estimado foi de R$ 8,3 milhões e um impacto de R$ 42,2 milhões em cinco anos (2013 a 2017). O período de tratamento considerado foi de 7,55 meses. Quanto ao erlotinibe, que foi incorporado no mesmo ano, o impacto orçamentário foi de R$ 45.240.885,00 para o SUS, considerando o acumulado nos cinco anos do horizonte de análise (2014 a 2018) (Ministério da Saúde 2013a, Ministério da Saúde, 2013b). Há de se considerar que a diferença para os resultados atuais se deve a não ter sido realizada uma correção dos valores para o ano em curso.

